# Congenital coronary artery-left ventricular multiple micro-fistulas and hypertrophic cardiomyopathy: a case report and literature review

**DOI:** 10.1186/s12872-022-02926-w

**Published:** 2022-11-12

**Authors:** Yue Liu, Zhiyuan Wang, Hong Zeng, Sibao Yang, Xiangdong Li

**Affiliations:** 1 Cardiology department, China-Japan Union Hospital of Jilin University, Changchun, 130033 China; 2 Jilin Provincial Key Laboratory for Genetic Diagnosis of Cardiovascular Disease, Changchun, 130033 China; 3 Jilin Provincial Cardiovascular Research Institute, 126 Xiantai Street, Changchun, 130033 Jilin Province China; 4 Ultrasound Department, China-Japan Union Hospital of Jilin University, Changchun, 130033 China

**Keywords:** Congenital coronary artery-left ventricular multiple micro-fistulas, Hypertrophic cardiomyopathy, Case report

## Abstract

**Background:**

Coronary artery-left ventricular multiple micro-fistulas (CA-LVMMFs) is a rare congenital vascular anomaly that may present with no obvious clinical symptoms or a typical angina attack. CA-LVMMFs is usually found unexpectedly during coronary angiography (CAG).

**Case presentation:**

We report a case of a 65-year-old man admitted to the hospital with acute coronary syndrome. CA-LVMMFs was found during coronary angiography. Echocardiography showed apical hypertrophy and blood flow signals were seen in the apical myocardium, connected with the left ventricle. We searched the MEDLINE database and found 39 relevant reports. We made statistics on the clinical characteristics of these patients and found half involved hypertrophy or perfusion defects in the ventricular septum or apex.

**Conclusion:**

As a rare congenital anomaly, the effect of CA-LVMMFs on patients is unclear. By reporting a case and summarizing literature reports, we found that CA-LVMMFs may be associated with myocardial hypertrophy, especially ventricular septal and apical hypertrophy.

**Supplementary Information:**

The online version contains supplementary material available at 10.1186/s12872-022-02926-w.

## Background

Coronary artery fistula (CAF) is a congenital vascular anomaly that occurs in 0.13%-0.22% of adults undergoing coronary angiography (CAG) [1]. There are two main types of CAF. The first type is isolated coronary fistula, which involves an abnormal connection between the coronary artery and any part of the heart chamber, pulmonary circulation, or systemic circulation. These fistulas can be distinguished by identifying the origin, destination, and path, and they occur in up to 90% of all CAF cases. The second type is congenital coronary artery-left ventricular multiple micro-fistulas (CA-LVMMFs), which is characterized by multiple small-caliber fistulas that can cause ventricular cavity opacity and is relatively rare. According to Salah's report, CA-LVMMFs occurs in 0.09 % [2] of adults undergoing CAG. We report a case of CA-LVMMFs diagnosed by CAG and describe their angiographic features. We also searched the MEDLINE database and found 39 cases reporting similar anatomical structures. We have summarized the similarities in these cases to provide a new perspective for understanding CA-LVMMFs.

## Case presentation

**Fig. 1:**
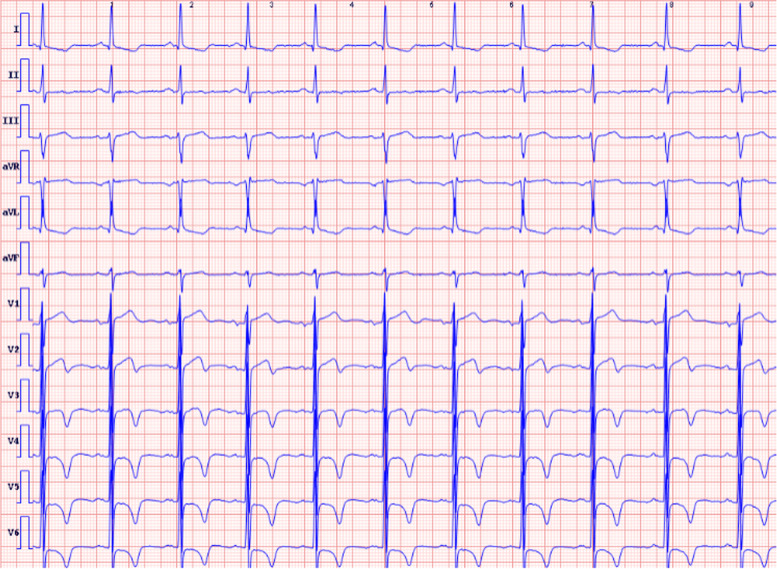
The resting electrocardiogram showed T-wave inversion in V2-V6 of the precordial leads.

**Fig. 2:**
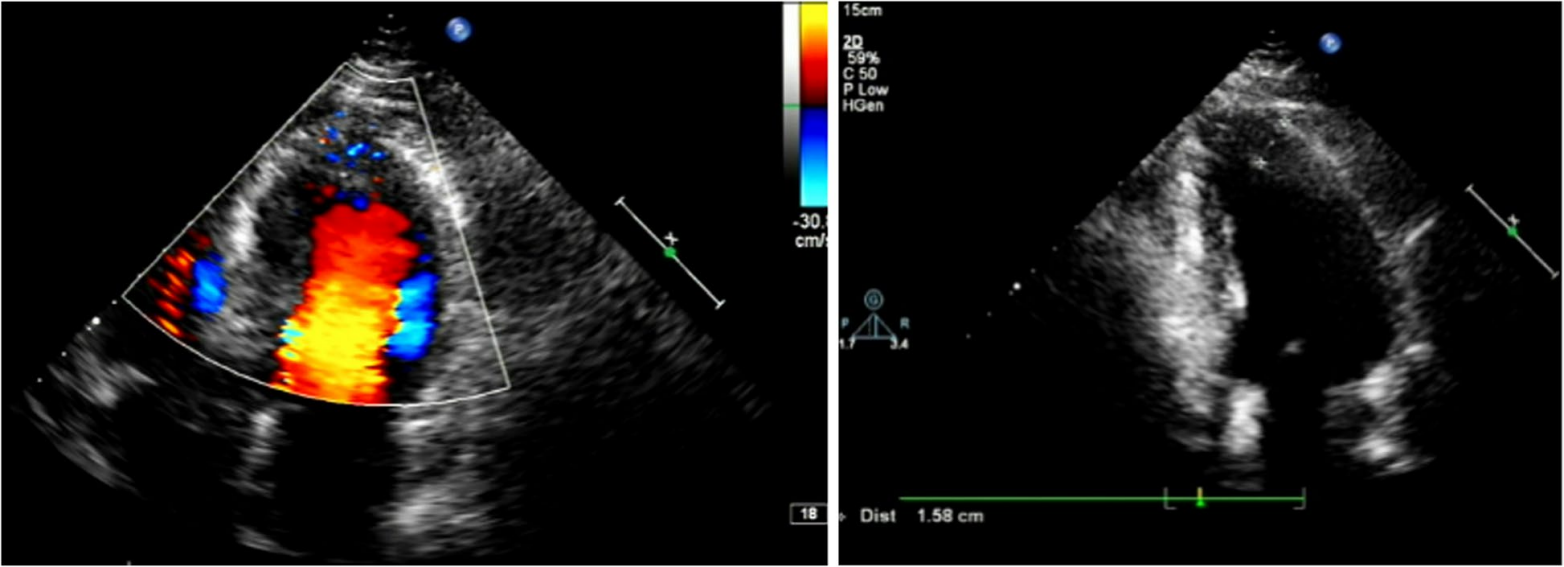
Abnormal manifestations of the patient's echocardiography. (B-1) Color-flow Doppler Imaging showed the blood flow signal was visible in the apical segment wall(yellow triangle), which was more obvious in the subepicardial, and the subendocardial blood flow was connected to the left ventricle. (B-2). Apical myocardium markedly thickened.

**Fig. 3:**
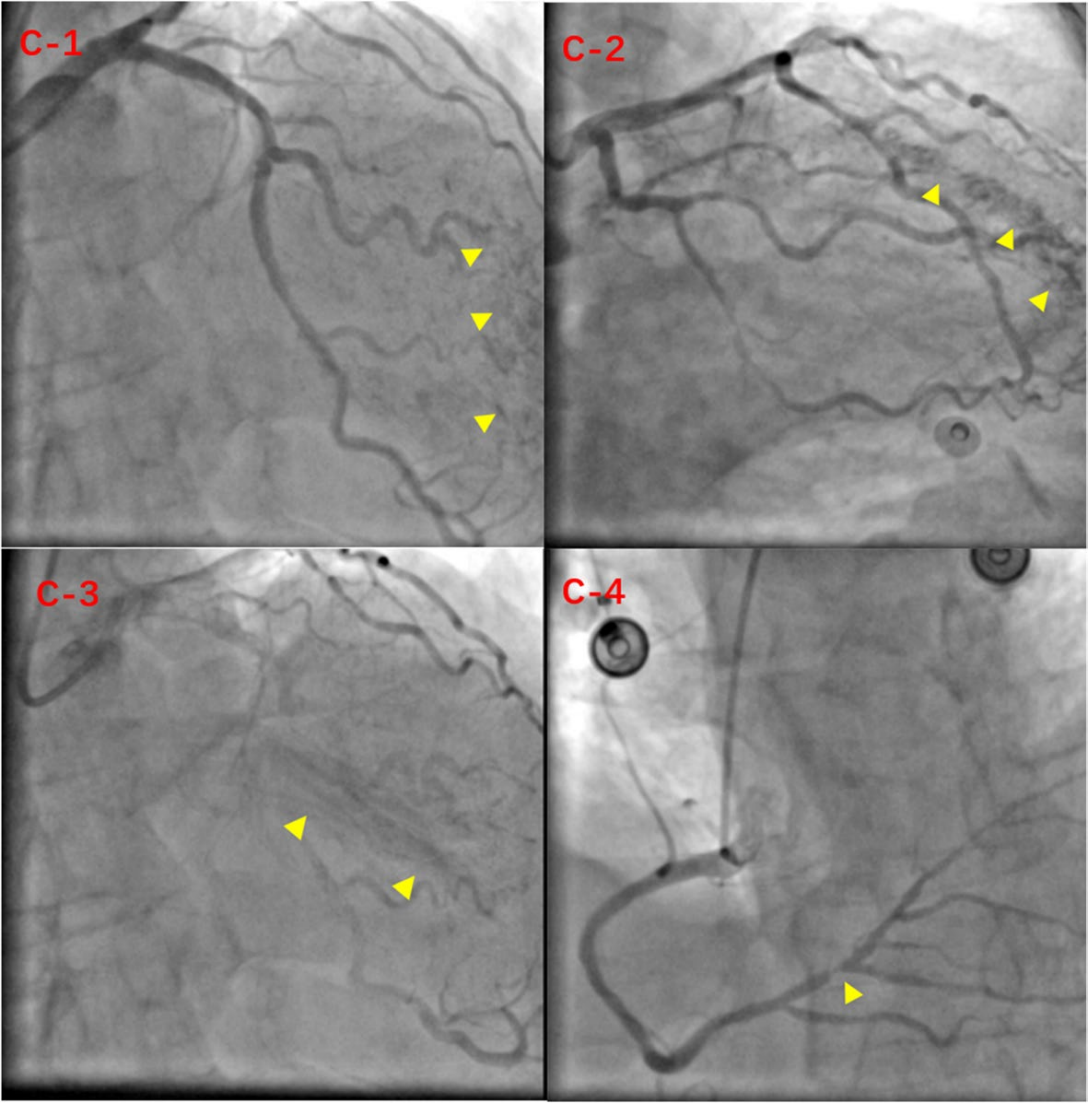
Abnormal manifestations of coronary angiography. During the left coronary angiography, the contrast agent entered from the diagonal branch (C-1, yellow triangle), rapidly flowed into the left ventricle via a diffuse plexus of multiple intramyocardial fistulas (C-2, yellow triangle), and completely turbidized the left ventricle. Subsequently, the contrast agent was discharged from the left ventricle during systole (C-3, yellow triangle). (C-4) There was severe stenosis in the distal of the RCA.

**Fig. 4:**
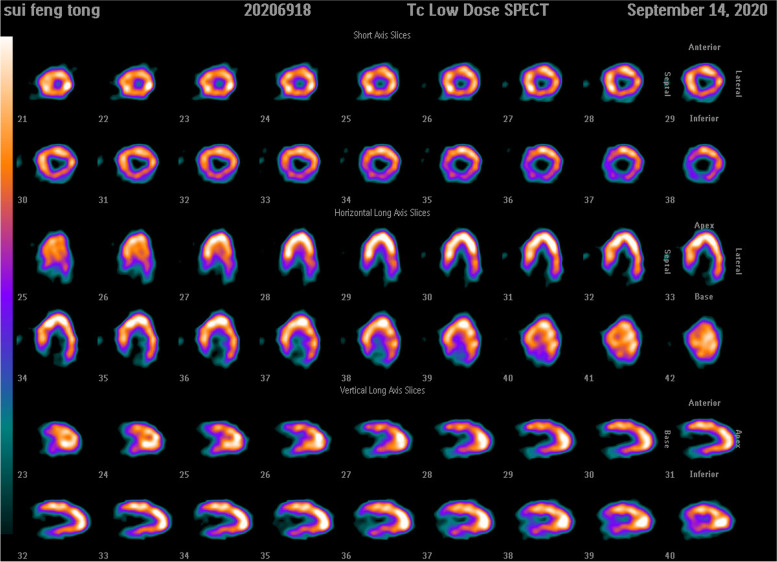
ECT showed that uneven distribution of 99mTC-MIBI in the myocardium and perfusion defect in the apical myocardium.

**Fig. 5:**
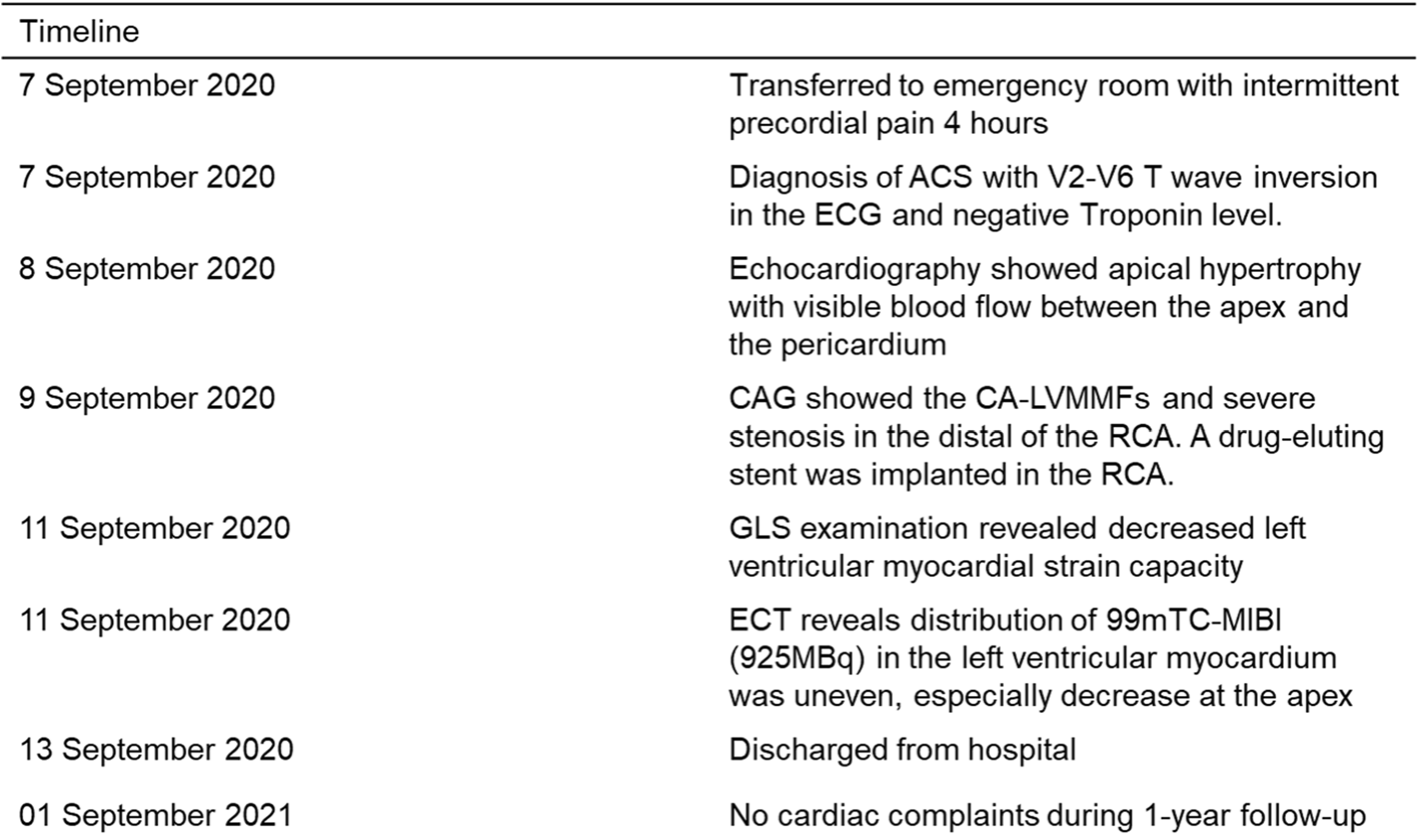
Timeline of the patient’s entire medical process.

A 65-year-old man was hospitalized with experiencing retrosternal pain for 4 h on September 7th, 2020. His past medical history included a 5-year history of hypertension and no history of diabetes mellitus. This was his first heart attack, which had never happened before. Physical examination revealed a blood pressure of 145/78 mmHg, a heart rate of 72 beats per minute, and a systolic murmur of 2/6 degrees in the apical and aortic auscultation areas. The resting electrocardiogram (Fig. 1) showed the T-wave inversion in V2-V6 of the precordial leads, but the Troponin I was negative and the NT-proBNP was 469 pg/ml (reference value range: 0-125). Echocardiography showed a normal-sized left ventricle with a thickened left ventricular wall, especially in the apical region up to 20 mm in thickness. The blood flow signal was visible in the apical segment wall, which was more obvious in the subepicardial, and the subendocardial blood flow was connected to the left ventricle (Fig. 2). Left ventricular systolic function was normal (EF=77%), with moderate mitral and aortic regurgitation (mitral regurgitation area 4.5 cm^2^; aortic regurgitation area 4.7 cm^2^). He then received a CAG examination, which showed that during the left coronary angiography, the contrast agent entered from the diagonal branch, and rapidly flowed into the left ventricle via a diffuse plexus of multiple intramyocardial fistulas, and completely turbidized the left ventricle. Subsequently, the contrast agent was discharged from the left ventricle during systole (Fig. 3. 1-3). An additional movie file showed this in more detail (see Additional file 1). There was severe stenosis in the distal of the right coronary artery (RCA) (Fig. 4. 4) . We performed drug-eluting stent implantation on the distal right coronary artery, followed by dual antiplatelet, statin, and beta-blocker therapy, and his angina symptoms were completely resolved. To further examine the effect of CAF on the myocardium, we performed myocardial strain examination with global longitudinal strain (GLS) measurements, which showed decreased left ventricular myocardial strain capacity (significantly decreased hypertrophic myocardial strain), decreased left ventricular wall synchrony, GLS of -12% (reference value, ≤-18%), and peak strain dispersion (PSD) of 111 ms (reference value, <45 ms). We also performed emission computed tomography (ECT) on the patient and found that the radiation distribution of 99mTC-MIBI (925MBq) in the left ventricular myocardium was uneven, especially decrease at the apex, which was consistent with the area supplied by the left anterior descending artery (Fig. 4), independent of stenosis of distal to the right coronary artery. Regretfully, the patient did not received further cardiac magnetic resonance due to financial reasons. Then he discharged on September 13th, 2020， and during the 12-month follow-up, there were no cardiovascular events occurred. Figure 5 shows the timeline of the patient’s entire medical process.

## Discussion and conclusion

We presented a case of CA-LVMMFs that showed interventricular septum and apex hypertrophy, and the ECT results suggested a myocardial perfusion defect in the anterior wall. A causal relationship between CA-LVMMFS and myocardial hypertrophy or perfusion defects has not been determined. By searching the MEDLINE database, we identified 39 patients [3–33] with similar anatomical structures and summarized their characteristics, as shown in Table 1 (see Additional file 2).

In the study, the mean age of the patients was 58.8 years, while 13 patients were male (33.3%) and 26 were female (66.7%). Twenty-five patients (64.1%) had fistulas from three coronary arteries, and 35 patients (89.7%) showed fistula communication with the left ventricle. Thirteen patients (33.3%) had a clear history of hypertension, 19 (48.7%) showed definite evidence of myocardial hypertrophy (including any position of the myocardium), 17 (43.6%) showed evidence of myocardial perfusion defects (including any location of the myocardium), 19 (48.7%) showed evidence of myocardial hypertrophy or myocardial perfusion defect (involving the ventricular septum or apex of the heart), and five had a definite diagnosis of hypertrophic cardiomyopathy (12.8%). The diagnostic criteria of the left ventricular wall or ventricular septal thickness of echocardiography were for hypertrophic cardiomyopathy ≥15mm and myocardial hypertrophy ≥12mm. Based on the above data, CA-LVMMFs cases included a high proportion of myocardial hypertrophy or myocardial perfusion defects, and the proportion of patients showing involvement of the ventricular septum or apex position was high. To exclude the interference of secondary myocardial hypertrophy caused by hypertension, we obtained the data for 26 patients without a history of hypertension and identified 11 patients with myocardial hypertrophy or perfusion defects, indicating that their myocardial hypertrophy and perfusion defects were unrelated to hypertension. To exclude the interference of myocardial perfusion defects caused by coronary artery stenosis, we obtained the data for 35 patients without coronary artery stenosis and 17 patients who showed evidence of myocardial perfusion defects (including any location of the myocardium), indicating that their myocardial perfusion defects were unrelated to coronary artery stenosis. We conclude that there may be an unrecognized association between CA-LVMMFs and myocardial hypertrophy and perfusion defects, especially in the ventricular septum and the apex of the heart.

Few reports have described the relationship between CA-LVMMFs and myocardial hypertrophy. We have two hypotheses: the first is that CA-LVMMFs initially develops abnormally in the coronary arteries, with increased blood flow directly into the left ventricle leading to increased volume loading and thus myocardial hypertrophy. The other is that the myocardial hypertrophy mediated by genetic mutations appears first and interferes with the development of coronary arteries in the embryo, which affects the microenvironment of vascular dedifferentiation and leads to the formation of CA-LVMMFs.

Hypertrophic cardiomyopathy (HCM) is defined by a wall thickness ≥ 15 mm in one or more LV myocardial segments measured by any imaging technique. In up to 60% of adolescents and adults with HCM, the disease is an autosomal dominant disorder caused by mutations in the cardiac sarcomeric protein genes. Mayo Clinic offers a simple clinically applicable phenotype-derived score that is a good predictor of a positive HCM genetic test result. The scoring system includes echocardiographic reverse morphological subtypes, age at diagnosis less than 45 years, maximum left ventricular wall thickness 20 mm or greater, and son on. The Toronto scoring system based on clinical characteristics and echocardiographic variables, including age, female gender, arterial hypertension, and so on， is also a good predictor of the probability of a positive genotype. In the early stage of HCM, patients are usually asymptomatic, and conventional noninvasive cardiac function indicators are within the normal range. As the disease progresses, LV diastolic and systolic function declines, and severe LV diastolic dysfunction develops. In patients with symptomatic left ventricular outflow tract obstruction, symptoms can be improved with medication, surgery, or alcohol ablation. Patients refractory to medical therapy may be candidates for heart transplantation [34].

The literature [35] shows that CA-LVMMFs were found in some patients with HCM after surgical resection. This suggests that hypertrophic cardiomyopathy may be associated with the abnormal development of similar micro-vessels, but most of these abnormal micro-vessels do not break through the hypertrophic myocardium and were exposed after surgery. But not all hypertrophic cardiomyopathy patients have CA-LVMMFs, possibly because the genetic mutations that cause hypertrophic cardiomyopathy are so diverse that only some of them interfere with coronary artery development. Biopsies of surgically resected hypertrophic myocardium may reveal clues, and genetic screening could be used in the future to identify these patients for abnormal genetic changes.

There is no consensus regarding the treatment of CA-LVMMFs. Due to the diffuse characteristics of abnormal microvascular fistulas, scholars have proposed conservative treatment and considered the need to prevent infective endocarditis. Both beta-blockers [14] and calcium channel blockers [15] have been reported to be effective, probably by improving the myocardial oxygen supply-and-demand mismatch, and ivabradine may be administered if these drugs are contraindicated [13]. Most patients show a good prognosis; conservative treatment can effectively relieve the symptoms, and few patients have an acute myocardial infarction, heart failure, and other complications.

In conclusion, as a rare congenital anomaly, the effect of CA-LVMMFs on patients is unclear. By reporting a case and summarizing literature reports, we found that CA-LVMMFs may be associated with myocardial hypertrophy, especially ventricular septal and apical hypertrophy.

## Supplementary Information

**Additional file 1: Video 1** Left coronary angiography in right anterior oblique 30° + cranial 20°2. **Video 2** Left coronary angiography in right anterior oblique 30° + caudal 20°.

**Additional file 2: Table1**

## Data Availability

Not applicable.

## Abbreviations

CA-LVMMFs: Coronary artery-left ventricular multiple micro-fistulas
CAG: Coronary angiography
CAF: Coronary artery fistula
ECT: Emission computed tomography
GLS: Global longitudinal strain
HCM: Hypertrophic cardiomyopathy
PSD: Peak strain dispersion
RCA: Right coronary artery
