# Correction: Congenital coronary artery-left ventricular multiple micro-fistulas and hypertrophic cardiomyopathy: a case report and literature review

**DOI:** 10.1186/s12872-022-02957-3

**Published:** 2022-11-30

**Authors:** Yue Liu, Zhiyuan Wang, Hong Zeng, Sibao Yang, Xiangdong Li

**Affiliations:** 1 Cardiology Department, China-Japan Union Hospital of Jilin University, Changchun, 130033 China; 2 Jilin Provincial Key Laboratory for Genetic Diagnosis of Cardiovascular Disease, Changchun, 130033 China; 3 Jilin Provincial Cardiovascular Research Institute, 126 Xiantai Street, Changchun, 130033 Jilin Province China; 4 Ultrasound Department, China-Japan Union Hospital of Jilin University, Changchun, 130033 China

**Correction to: BMC Cardiovascular Disorders (2022) 22:483** 10.1186/s12872-022-02926-w

Following the publication of the original article [1], the Fig. 4 has been modified, and the same is shown below:

**Fig. 4:**
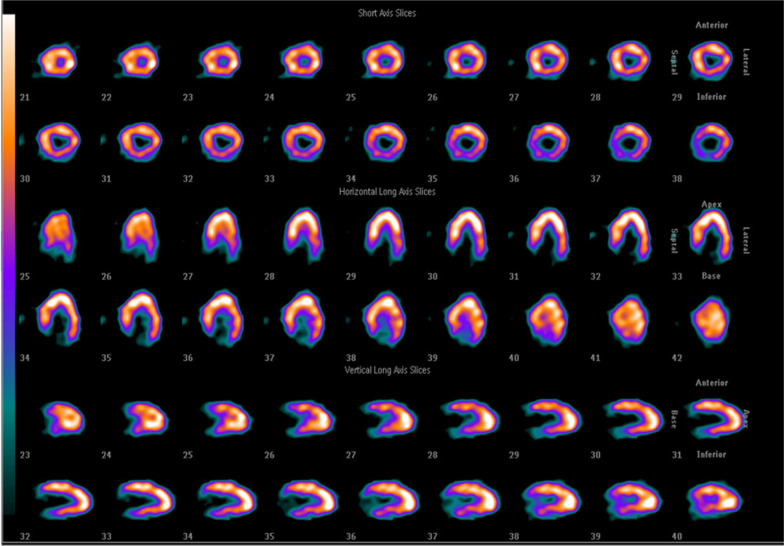
ECT showed that uneven distribution of 99mTC-MIBI in the myocardium and perfusion defect in the apical myocardium

The original article has been corrected.
